# Stage-Specific Inhibition of MHC Class I Presentation by the Epstein-Barr Virus BNLF2a Protein during Virus Lytic Cycle

**DOI:** 10.1371/journal.ppat.1000490

**Published:** 2009-06-26

**Authors:** Nathan P. Croft, Claire Shannon-Lowe, Andrew I. Bell, Daniëlle Horst, Elisabeth Kremmer, Maaike E. Ressing, Emmanuel J. H. J. Wiertz, Jaap M. Middeldorp, Martin Rowe, Alan B. Rickinson, Andrew D. Hislop

**Affiliations:** 1 School of Cancer Sciences, University of Birmingham, Edgbaston, Birmingham, United Kingdom; 2 Department of Medical Microbiology, Leiden University Medical Center, Leiden, The Netherlands; 3 Institute of Molecular Immunology, Helmholtz Zentrum München, München, Germany; 4 Department of Medical Microbiology, University Medical Centre Utrecht, Utrecht, The Netherlands; 5 Department of Pathology, VU University Medical Centre, Amsterdam, The Netherlands; University of Wisconsin-Madison, United States of America

## Abstract

The gamma-herpesvirus Epstein-Barr virus (EBV) persists for life in infected individuals despite the presence of a strong immune response. During the lytic cycle of EBV many viral proteins are expressed, potentially allowing virally infected cells to be recognized and eliminated by CD8^+^ T cells. We have recently identified an immune evasion protein encoded by EBV, BNLF2a, which is expressed in early phase lytic replication and inhibits peptide- and ATP-binding functions of the transporter associated with antigen processing. Ectopic expression of BNLF2a causes decreased surface MHC class I expression and inhibits the presentation of indicator antigens to CD8^+^ T cells. Here we sought to examine the influence of BNLF2a when expressed naturally during EBV lytic replication. We generated a BNLF2a-deleted recombinant EBV (ΔBNLF2a) and compared the ability of ΔBNLF2a and wild-type EBV-transformed B cell lines to be recognized by CD8^+^ T cell clones specific for EBV-encoded immediate early, early and late lytic antigens. Epitopes derived from immediate early and early expressed proteins were better recognized when presented by ΔBNLF2a transformed cells compared to wild-type virus transformants. However, recognition of late antigens by CD8^+^ T cells remained equally poor when presented by both wild-type and ΔBNLF2a cell targets. Analysis of BNLF2a and target protein expression kinetics showed that although BNLF2a is expressed during early phase replication, it is expressed at a time when there is an upregulation of immediate early proteins and initiation of early protein synthesis. Interestingly, BNLF2a protein expression was found to be lost by late lytic cycle yet ΔBNLF2a-transformed cells in late stage replication downregulated surface MHC class I to a similar extent as wild-type EBV-transformed cells. These data show that BNLF2a-mediated expression is stage-specific, affecting presentation of immediate early and early proteins, and that other evasion mechanisms operate later in the lytic cycle.

## Author Summary

Epstein-Barr virus (EBV) is carried by approximately 90% of the world's population, where it persists and is chronically shed despite a vigorous specific immune response, a key component of which are CD8^+^ T cells that recognize and kill infected cells. The mechanisms the virus uses to evade these responses are not clear. Recently we identified a gene encoded by EBV, BNLF2a, that when expressed ectopically in cells inhibited their recognition by CD8^+^ T cells. To determine the contribution of BNLF2a to evasion of EBV-specific CD8^+^ T cell recognition and whether EBV encoded additional immune evasion mechanisms, a recombinant EBV was constructed in which BNLF2a was deleted. We found that cells infected with the recombinant virus were better recognized by CD8^+^ T cells specific for targets expressed co-incidently with BNLF2a, compared to cells infected with a non-recombinant virus. However, proteins expressed at late stages of the viral infection cycle were poorly recognised by CD8^+^ T cells, suggesting EBV encodes additional immune evasion genes to prevent effective CD8^+^ T cell recognition. This study highlights the stage-specific nature of viral immune evasion mechanisms.

## Introduction

The detection and elimination of virally infected cells by the host immune system relies heavily upon CD8^+^ T cells recognizing peptides endogenously processed and presented by HLA class I molecules. Proteasomal degradation of endogenously synthesized proteins provides a source of peptides which are delivered into the endoplasmic reticulum by the transporter associated with antigen processing (TAP), where they are loaded onto nascent HLA-class I molecules. Peptide:HLA-class I complexes are then transported to the cell surface where CD8^+^ T cells examine these complexes with their T cell receptors. Recognition of these complexes leads to the killing of the infected cell by the CD8^+^ T cell (reviewed in [1],[2]).

As such, many viruses have developed strategies to evade CD8^+^ T cell recognition in order to aid their transmission and persistence within hosts. This is particularly true for the herpesviruses; large double-stranded DNA viruses characterized by their ability to enter a latent state within specialized cells in their respective hosts, with this itself a form of immune evasion due to the transcriptional silencing of most if not all genes. However, herpesviruses occasionally undergo reactivation into their lytic cycle, where a large number of viral genes are expressed. Here there is a sequential cascade of gene expression beginning with the immediate early genes, followed by the early genes and finally the late genes. Potentially then many targets for CD8^+^ T cell recognition are generated during lytic cycle replication. The finding of immune evasion mechanisms in members of each of the three α-, β- and γ-herpesvirus subfamilies highlights the strong immunological pressure these viruses are under. These evasion strategies often subvert cellular processes involved in the generation and presentation of epitopes to T cells (reviewed in [3],[4]). The importance of these processes is highlighted by the convergent evolution seen in herpesviruses, where members of the different subfamilies target the same points involved in the generation of CD8^+^ T cell epitopes but use unrelated proteins to do this.

Until recently, less evidence has been available on immune evasion by the lymphocryptoviruses (LCV, γ1-herpesviruses) during lytic cycle. The prototypic virus of this genus, Epstein-Barr virus (EBV), infects epithelial cells and B lymphocytes, establishing latency in the latter cell type. Central to EBV's biology is its ability to expand the reservoir of latently infected B cells through growth-transforming gene expression, independent of lytic replication [5]. It was unclear then whether lytic immune evasion mechanisms would be required by EBV to amplify the viral reservoir within a host. However, during lytic cycle replication, presentation of EBV epitopes to cognate CD8^+^ T cells falls with the progression of the lytic cycle, while B cells replicating EBV have decreased levels of surface HLA-class I and decreased TAP function [6]–[8]. These observations suggested that EBV interferes with antigen processing during lytic cycle replication. Targeted screening of EBV genes for immune evasion function led to the identification of the early expressed lytic cycle gene *BNLF2a* which functions as a TAP inhibitor [9]. This novel immune evasion gene encodes for a 60 amino acid protein that disrupts TAP function by preventing both peptide- and ATP-binding to this complex. Consequently, cells expressing BNLF2a *in vitro* show decreased surface HLA-class I levels and are refractory to CD8^+^ T cell killing when co-expressed with target antigens [9].

In the current study we analyze the influence BNLF2a has on presentation of EBV-specific epitopes during lytic cycle replication, to determine whether BNLF2a acts alone or whether other immune evasion mechanisms are present in EBV and how BNLF2a affects antigen presentation during the different phases of gene expression. The impact of BNLF2a was isolated through the construction of a recombinant EBV lacking the gene and this virus used to infect cells for antigen processing and presentation studies. Cells replicating this *BNLF2a*-deleted virus were found to be better recognized by immediate early and early antigen-specific CD8^+^ T cells but not late antigen-specific T cells. Consistent with this finding, surface class I HLA expression was restored to normal levels in cells expressing immediate early but not late expressed EBV proteins. Our results suggest that immune evasion mechanisms in addition to BNLF2a are operational during EBV lytic cycle replication.

## Results

### Construction of a ΔBNLF2a mutant virus

**Figure 1:**
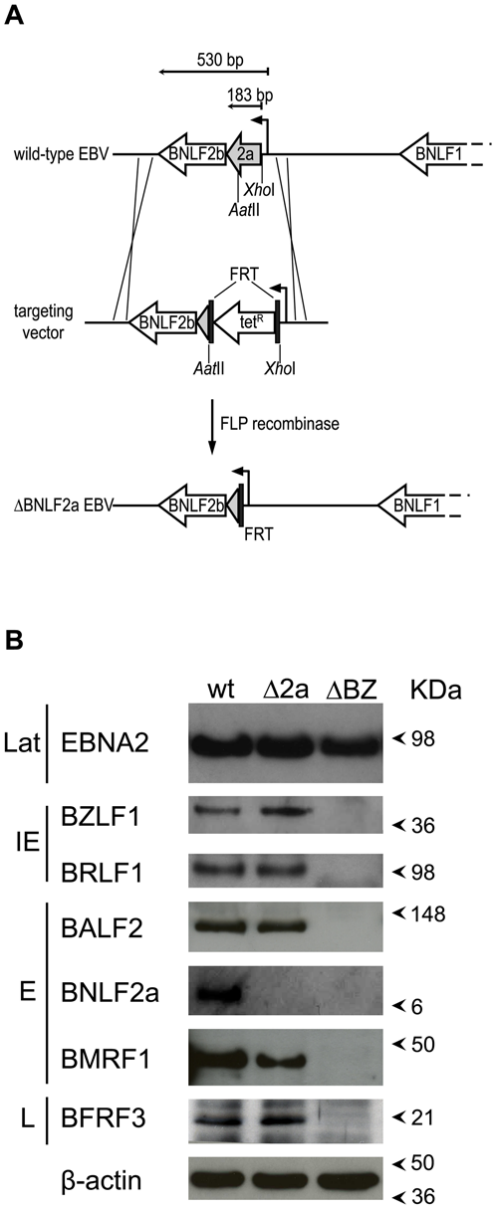
Generation of a mutant Epstein-Barr virus deleted for BNLF2a (ΔBNLF2a). (A) Schematic drawing of the *BNLF2a*-containing region of the EBV genome, before and after disruption of the *BNLF2a* open reading frame. Removal of the tetracycline resistance cassette by flp recombinase leaves one flp recombinase target (*FRT*) site intact. (B) LCLs transformed with either the wild-type (wt), ΔBNLF2a (Δ2a) or ΔBZLF1 (ΔBZ) viruses were analysed by Western blot for expression of BNLF2a, several representative lytic cycle antigens, and the latent cycle expressed protein EBNA2. Antibodies specific for β-actin were used to ensure equal protein loading. Lat, latent; IE, immediate early; E, early; L, late.

We initially disrupted the *BNLF2a* gene of the B95.8 strain of EBV contained within a BAC by insertional mutagenesis (Figure 1A). A targeting plasmid was created in which the majority of the *BNLF2a* gene was replaced with a tetracycline resistance cassette which in turn was flanked by FLP recombinase target (*FRT*) sites. This vector was recombined with the EBV BAC and recombinants selected. Such clones, designated ΔBNLF2a, had the tetracycline gene removed by FLP recombinase and were screened for deletion of the *BNLF2a* gene by restriction endonuclease analysis and sequencing (data not shown). ΔBNLF2a BACs were then stably transfected into 293 cells and virus replication induced by transfection of a plasmid encoding the EBV lytic switch protein BZLF1. Virus was also produced from cells transduced with the wild-type B95.8 EBV BAC and a B95.8 EBV BZLF1-deleted BAC (ΔBZLF1) [10], encoding a virus unable to undergo lytic cycle replication unless BZLF1 is supplied in trans.

The different recombinant EBVs derived from the 293 cells were used to transform primary B cells, to establish lymphoblastoid cell lines (LCLs). To determine if expression of other viral proteins was affected by the deletion of BNLF2a, western blot analysis on lysates of LCLs generated from wild-type, ΔBNLF2a and ΔBZLF1 viruses was performed. As a subset of cells in the LCL culture will spontaneously enter lytic cycle replication, blots were probed with antibodies specific for representative proteins expressed during lytic cycle as well as latent cycle expressed proteins. Figure 1B shows typical blots of lysates probed for the immediate early proteins BZLF1 and BRLF1, the early proteins BALF2, BNLF2a and BMRF1, the late protein BFRF3 and the latent protein EBNA2. No difference in expression of these proteins was observed between the wild-type and ΔBNLF2a virus transformed LCLs, with the exception of BNLF2a protein which was not present as expected in ΔBNLF2a LCLs. No lytic cycle protein expression could be detected in ΔBZLF1 LCLs.

### Deletion of BNLF2a confers an increase in immediate early and early antigen recognition by cognate CD8^+^ T cells, but has no effect on late antigen recognition

**Figure 2:**
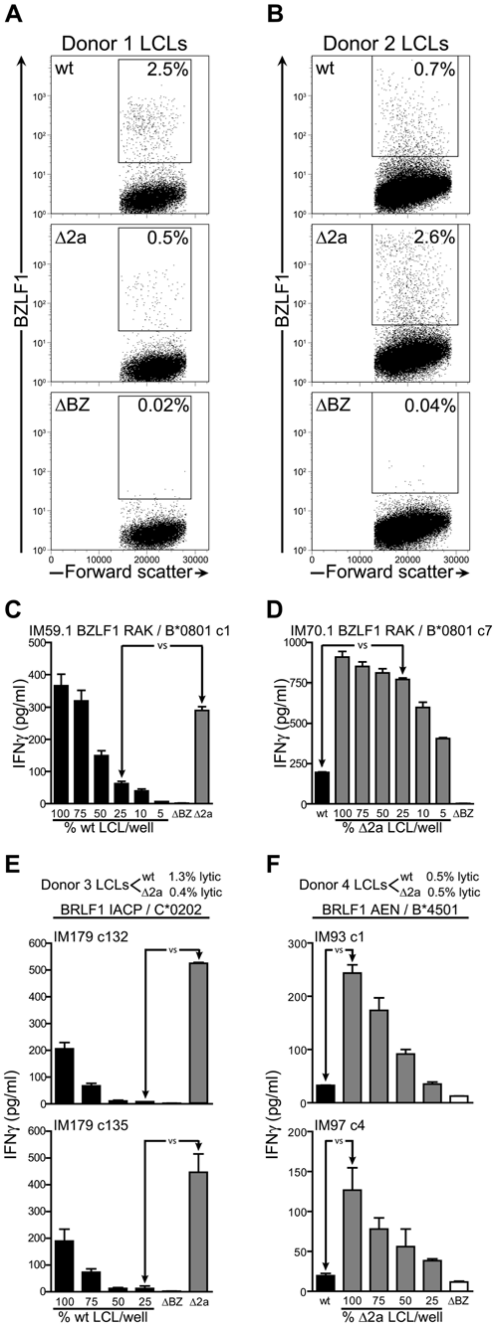
Estimation of ΔBNLF2a and wild-type LCLs expressing lytic antigens; recognition by immediate early antigen-specific CD8^+^ T cells. The proportion of LCLs spontaneously reactivating into lytic cycle was assessed by intracellular BZLF1 staining and analysis by flow cytometry, with representative examples shown for LCLs derived from two different donors: (A) donor 1 and (B) donor 2. Immediate early lytic cycle CD8^+^ T cell recognition of wild-type (wt), ΔBNLF2a (Δ2a) and ΔBZLF1 (ΔBZ) LCLs using HLA-B*0801-restricted RAK (BZLF1) clones against appropriately HLA matched donor 1 and 2 LCLs (C and D respectively) was measured by IFNγ ELISA. Results using wild-type or ΔBNLF2a cells diluted with ΔBZLF1 cells as appropriate are shown, where arrows indicate equivalent numbers of lytic antigen expressing cells. Experiments were also conducted using HLA-C*0202-restricted IACP (BRLF1) clones against donor 3 LCLs (E), and HLA-B*4501-restricted AEN (BRLF1) clones against donor 4 LCLs (F). For donor 4, both the wild-type and ΔBNLF2a LCLs were diluted with ΔBZLF1 LCL (wild-type-LCL titration data not shown). Data are represented as mean+/−SEM.

A panel of different donor derived LCLs transformed with wild-type, ΔBNLF2a, and ΔBZLF1 viruses were employed to study lytic antigen recognition by EBV lytic phase-specific CD8^+^ T cells. Here we planned to incubate these LCLs with the different types of lytic antigen-specific CD8^+^ T cells and assay for T cell recognition by IFN-γ secretion. However, the percentage of LCLs that spontaneously enter lytic cycle is variable. Initially then we quantified the number of cells within the LCL cultures expressing the lytic cycle marker BZLF1 by flow cytometry. Figures 2A and 2B show representative flow plots of wild-type, ΔBNLF2a and ΔBZLF1 LCLs stained for BZLF1 expression using LCLs derived from two donors. Typically we found between 0.5–3% of wild-type and ΔBNLF2a LCLs expressed BZLF1 (upper and middle panels), whilst none was observed in ΔBZLF1 LCLs (lower panels).

To ensure we used equivalent numbers of the different types of lytic antigen positive cells in our T cell recognition experiments, we developed a system to equalize the number of lytic antigen positive cells in each assay. Here the proportion of BZLF1 expressing cells in each culture were equalized by making a dilution series of the LCL with the highest percentage of BZLF1 expressing cells with the antigen negative ΔBZLF1 LCL derived from that donor. T cell recognition of the different LCL transformants was then measured by incubating these LCLs with CD8^+^ T cells specific for epitopes derived from proteins expressed in immediate early, early and late phases of the EBV lytic cycle and measuring IFNγ release by the T cells. We have previously shown that CD8^+^ T cells in these assays directly recognize lytically infected cells and not cells which have exogenously taken up antigen and re-presented it [6]. Figure 2C shows results of a T cell recognition experiment using LCL targets derived from donor 1. In this case the more lytic wild-type LCL was diluted with the ΔBZLF1 LCL to give equivalent numbers of lytic targets in the assay. When CD8^+^ T cells specific for the immediate early HLA-B*0801 restricted BZLF1 RAK epitope were incubated with the different LCLs, a 6-fold increase in recognition of the ΔBNLF2a LCL was observed compared to the wild-type LCL as measured by secretion of IFNγ. Similar results were obtained using LCLs derived from donor 2 (Figure 2D). In this case the more lytic ΔBNLF2a LCL was diluted with the antigen-negative ΔBZLF1 LCL. When the cultures were equalized for BZLF1 expression a 3-fold increase in recognition of the ΔBNLF2a LCL was seen when compared to recognition of the wild-type LCL.

A similar trend was observed for recognition of epitopes derived from the other immediate early protein BRLF1. Here CD8^+^ T cells specific for the HLA-C*0202 restricted epitope IACP (Figure 2E) and the HLA-B*4501 restricted epitope AEN (Figure 2F) were used to probe antigen presentation by the LCL sets derived from donors 3 and 4 respectively. As shown in Figures 2E and 2F, the ΔBNLF2a LCLs from both donors were recognized more efficiently than the wild-type LCL using both T cell specificities. The IACP clones showed a 50-fold increase and the AEN clones showed a 4–5-fold increase in IFNγ secretion upon challenge with the LCLs.

**Figure 3:**
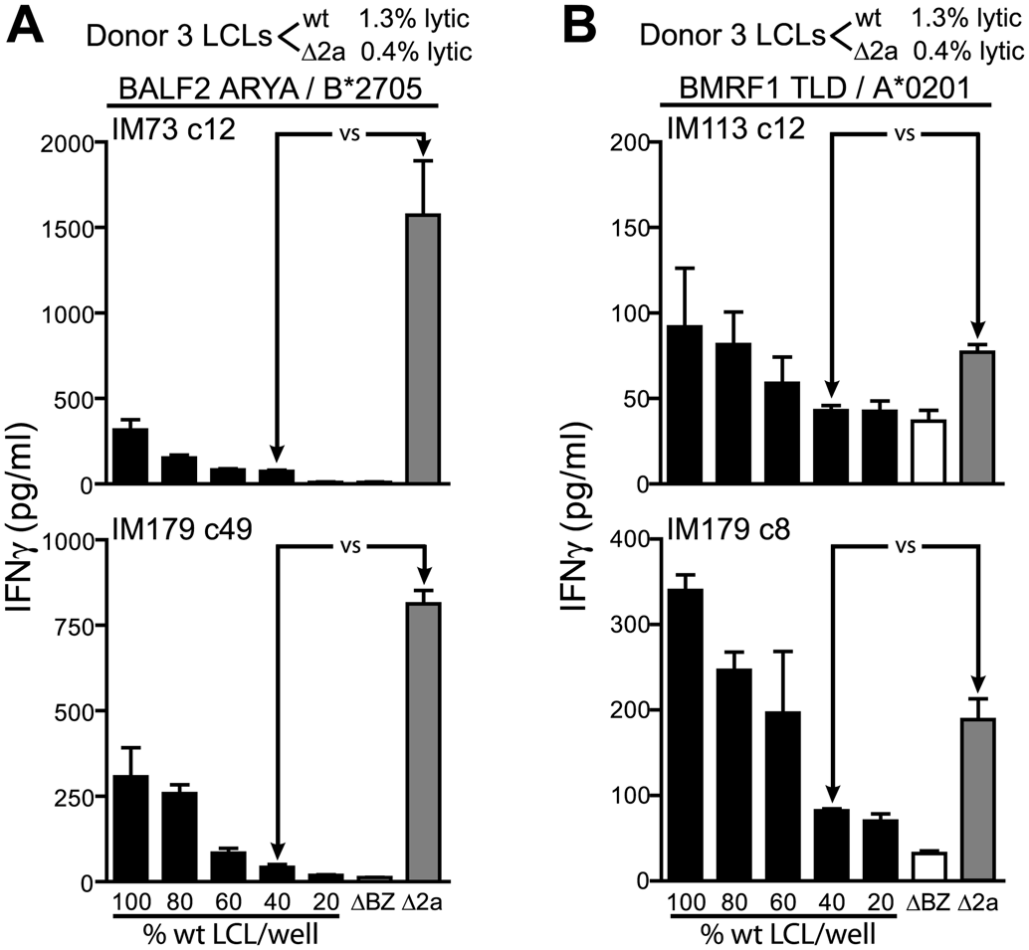
Recognition of ΔBNLF2a LCLs and wild-type LCLs by early antigen-specific CD8^+^ T cells. LCLs from donor 3 were measured for lytic antigen expression and the percentage positive indicated. The proportion of lytic antigen positive wild-type (wt) and ΔBNLF2a (Δ2a) cells were equalised by dilution with ΔBZLF1 (ΔBZ) LCL and recognition assays performed as described in Figure 2. Recognition of early lytic antigen targets was assessed using CD8^+^ T cells specific for the HLA-B*2705-restricted ARYA (BALF2) epitope (A) and the HLA-A*0201-restricted TLD (BMRF1) epitope (B). Arrows indicate equivalent numbers of lytic antigen expressing cells. Data are represented as mean+/−SEM.

We next measured recognition of the different LCL types using CD8^+^ T cells specific for two early antigens; the HLA-B*2705 restricted ARYA epitope from BALF2 and the HLA-A*0201 restricted TLD epitope from BMRF1. Here we tested multiple T cell clones derived from three donors against three different donor derived sets of LCLs. Figure 3 shows representative results using ARYA- and TLD-specific T cell clones against LCLs derived from donor 3. Similar to what was seen for the immediate early antigens, T cell recognition of the early antigens was increased upon challenge with the ΔBNLF2a LCL compared to the wild-type LCL, with the most potent increase in recognition observed using the BALF2-specific clones which showed a 20-fold increase in recognition (Figure 3A). The TLD epitope from BMRF1 was found to be recognized the poorest in these assays, never the less a two-fold increase in recognition of the ΔBNLF2a LCL compared to the wild-type was consistently observed using independently derived T cell clones and LCLs derived from different donors (Figure 3B). Multiple clones of a third early specificity, HLA-A*0201 BMLF1, also showed increased recognition of the ΔBNLF2a LCL (see below).

**Figure 4:**
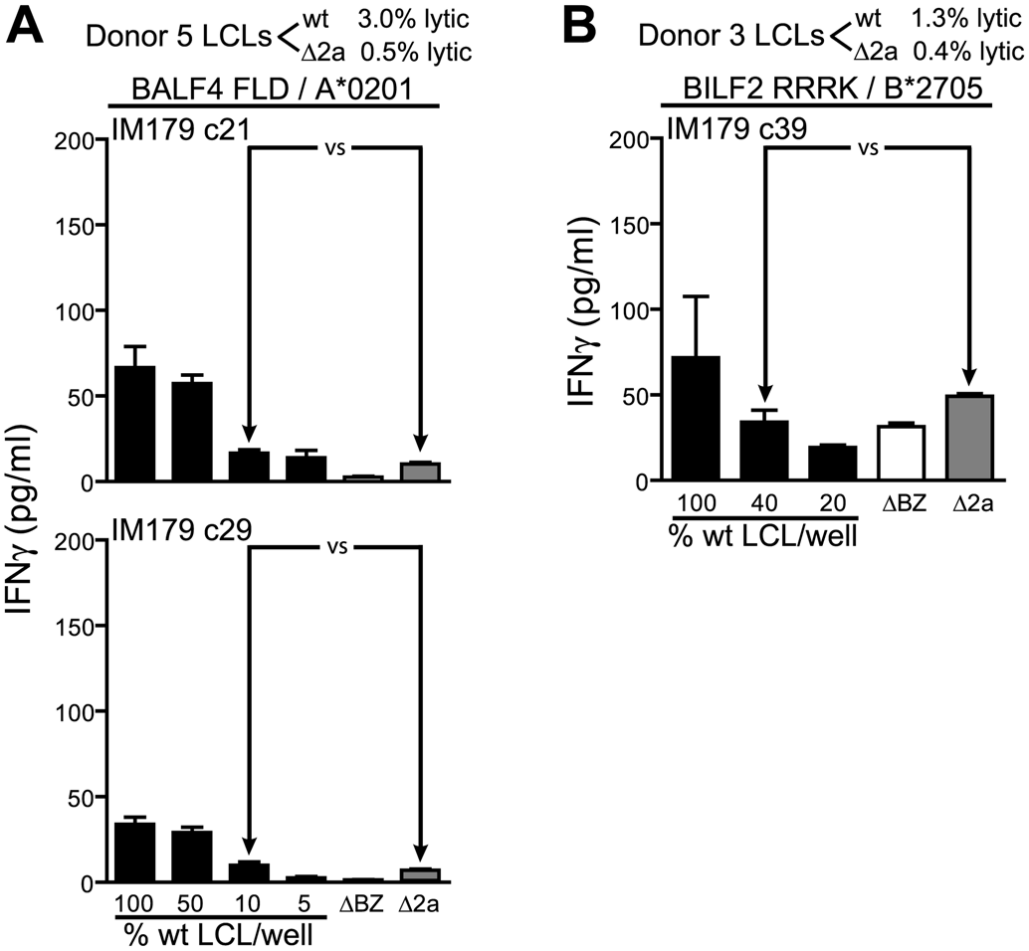
Recognition of ΔBNLF2a LCLs and wild-type LCLs by late antigen-specific CD8^+^ T cells. LCLs from donors 3 and 5 were measured for lytic antigen expression and the percentage positive indicated. The proportion of lytic antigen positive wild-type (wt) and ΔBNLF2a (Δ2a) cells were equalised by dilution with ΔBZLF1 (ΔBZ) LCL and recognition assays performed as described in Figure 2. Recognition of late lytic antigen targets was assessed using CD8^+^ T cells specific for the HLA-A*0201-restricted FLD (BALF4) epitope (A) and the HLA-B*2705-restricted RRRK (BILF2) epitope (B). Arrows indicate equivalent numbers of lytic antigen expressing cells. Data are represented as mean+/−SEM.

We next turned to study recognition of late-expressed antigens using T cells specific for the HLA-A*0201 restricted FLD epitope from BALF4 and the HLA-B*2705 restricted RRRK epitope from BILF2. We have found that these two epitopes are processed independently and dependently of the proteasome respectively, with the BALF4 epitope presented independently of TAP (data not shown). We would predict from our previous studies of TAP dependence of peptide-epitopes that the hydrophilic BILF2 peptide RRRK would be processed in a TAP dependent manner [11]. Figures 4A and 4B show representative results of experiments using two FLD-specific clones and one RRRK-specific clone assayed against two different donor derived LCLs. T cell recognition of late-expressing ΔBNLF2a and wild-type LCLs was found to be low but of an equivalent level. This pattern of recognition was seen using LCL sets derived from three other donors (data not shown).

**Figure 5:**
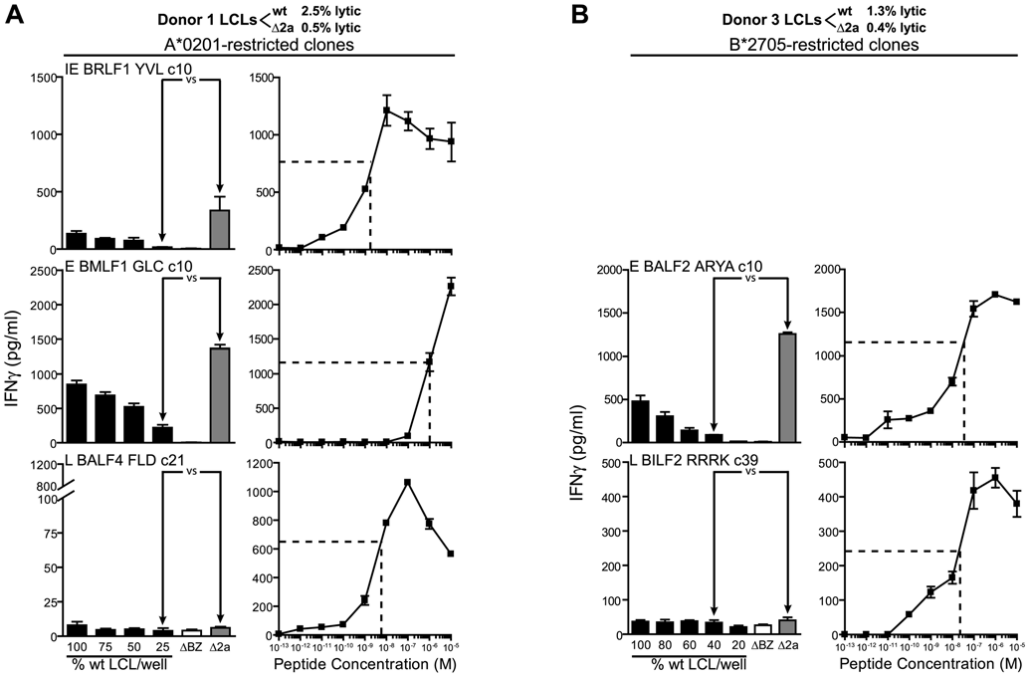
Comparative CD8^+^ T cell recognition of immediate early, early and late antigens expressed by ΔBNLF2a versus wild-type LCLs. (A) LCLs from donor 1 were measured for lytic antigen expression and the percentage positive indicated. The proportion of lytic antigen positive wild-type (wt) and ΔBNLF2a (Δ2a) cells were equalised by dilution with ΔBZLF1 (ΔBZ) LCL and recognition assays performed as described in Figure 2. Recognition of immediate early (IE), early (E) and late (L) lytic antigen targets was assessed in parallel using representative CD8^+^ T cells specific for the HLA-A*0201 restricted epitopes YVL (BRLF1), GLC (BMLF1) and FLD (BALF4) (left panels). Simultaneously, the functional avidity of these clones was measured by challenging the CD8^+^ T cells with ΔBZLF1 LCLs sensitized with 10-fold dilutions of the peptide epitope and the dose of peptide giving 50% maximal recognition determined (dashed line, right panels). (B) LCLs from donor 3 were measured for lytic antigen expression and the percentage positive indicated. The proportion of lytic antigen positive cells were equalised by dilution with ΔBZLF1 LCL and recognition assays performed as described in Figure 2. Recognition of early and late lytic antigen targets was assessed in parallel using representative CD8^+^ T cells specific for the HLA-B*2705 restricted epitopes ARYA (BALF2), and RRRK (BILF2) (left panels). Functional avidity of these clones was measured simultaneously as in (A). Arrows indicate equivalent numbers of lytic antigen expressing cells. Data are represented as mean+/−SEM.

To confirm the above results and minimize any variability between assays, we tested the recognition of the different LCL types in parallel by CD8^+^ T cell clones specific for epitopes that were presented by the same HLA molecule but produced at different phases in the replication cycle. Initially we compared recognition of the donor 1 set of LCLs by the HLA-A*0201 restricted CD8^+^ T cells specific for the YVL epitope from the immediate early protein BRLF1, the GLC epitope derived from the early expressed protein BMLF1 and the FLD epitope from the late expressed BALF4 protein. In LCLs made with the BNLF2a-deleted virus there was a clear increase in the ability of YVL- and GLC-specific CD8^+^ T cells to recognize these targets in comparison to the wild-type LCLs, with these specificities showing a 20- and 6-fold increase in IFN-γ secretion respectively (Figure 5A left panels). We also checked recognition in parallel with the HLA-A*0201 restricted TLD-specific clones which showed an increase in recognition similar to what we observed above (data not shown). By contrast, no apparent difference in recognition was observed using the CD8^+^ T cells specific for the late-derived FLD epitope. In parallel we also estimated the functional avidity of these T cell clones by IFNγ secretion in response to ΔBZLF1 LCLs loaded with 10-fold dilutions of epitope peptide (Figure 5A right panels). The 50% optimal recognition of the late effector FLD c21 was similar to that of the immediate early effector YVL c10, both being in the 10^−8^–10^−9^ M range of peptide avidity, whilst the early effector GLC c10 was less avid with a 50% optimal recognition of 10^−6^ M.

In a second series of experiments we compared the ability of the donor 3 set of LCLs to be recognized by HLA-B*2705 restricted CD8^+^ T cells. Here we used clones specific for the ARYA epitope derived from the early protein BALF2 and the RRRK epitope derived from the late protein BILF2. Again we found that the LCLs made using the BNLF2a-deleted virus were well recognized by the early antigen-specific effector compared to the wild-type transformed LCLs with a 14-fold increase in recognition (Figure 5B left panels), but both LCL types were recognized at an equivalent low level by the late-specific cells. In peptide titration assays the 50% optimal CD8^+^ T cell recognition values for the ARYA and RRRK clones were similar, at 4×10^−7^ and 2×10^−7^ respectively (Figure 5B right panels).

To confirm that the increased recognition of the ΔBNLF2a LCLs by the immediate early and early T cells seen in these experiments was due to the absence of BNLF2a and not to a secondary mutation within the ΔBNLF2a virus, we re-expressed BNLF2a in the ΔBNLF2a LCLs and conducted recognition assays on these cells. ΔBNLF2a LCLs were transfected with a BNLF2a expression vector which co-expressed the truncated nerve growth factor receptor (NGFR) and cells expressing this receptor selected with magnetic beads. These BNLF2a expressing cells and were used as targets in standard recognition assays alongside NGFR negative BNLF2a negative cells from the transfection, wild-type LCLs, unmanipulated ΔBNLF2a LCLs and ΔBZLF1 LCLs. T cells specific for the immediate early epitope AEN and early epitope ARYA were used as effectors in parallel assays. Figure S1 shows representative results of two independent transfection experiments. For both CD8^+^ T cell clones, re-expression of BNLF2a in the ΔBNLF2a LCLs decreased recognition of these LCLs to low levels relative to the unmanipulated ΔBNLF2a LCL, suggesting the increased recognition of the ΔBNLF2a LCLs observed in the previous experiments is due to the absence of BNLF2a.

### EBV BNLF2a is expressed during lytic cycle concomitant with peak immediate early and early gene expression

An unexpected outcome of the recognition experiments was the increased detection of immediate early antigens in the ΔBNLF2a transformed LCLs by the cognate CD8^+^ T cells. Immediate early genes are expressed prior to when the early gene *BNLF2a* would be expected to be expressed and so epitopes derived from immediate early proteins would not likely be well protected from presentation to CD8^+^ T cells. To clarify when *BNLF2a* is transcribed and expressed relative to the other genes of interest, we studied the transcription and protein expression kinetics of this gene and others that were used in our T cell recognition assays by qRT-PCR and western blot analysis during lytic replication. Here we used the EBV-infected AKBM cell line in which lytic EBV replication can be induced by cross-linking surface IgG receptors with anti-IgG antibodies [8] as a source of RNA and protein for analysis.

**Figure 6:**
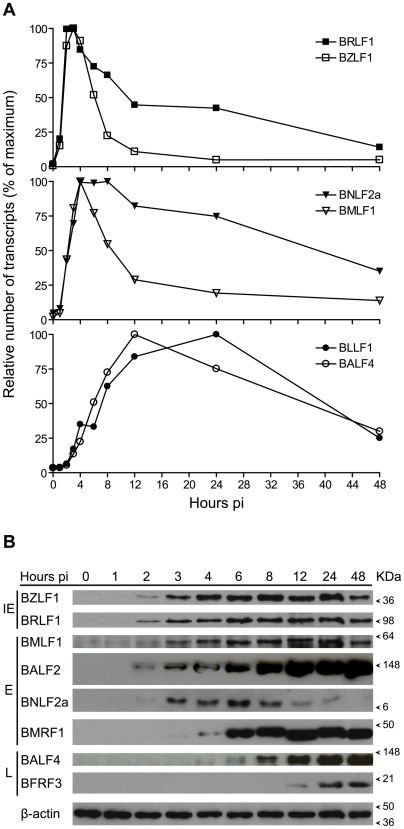
RNA and protein expression kinetics of BNLF2a relative to immediate early, early and late genes. AKBM cells containing latent virus were stimulated to induce lytic cycle replication, samples harvested at the indicated times and selected viral transcript and protein levels estimated. Samples were harvested from 0 to 48 hours post induction (pi), and RNA was harvested and subjected to qRT-PCR detection of *BZLF1*, *BRLF1*, *BMLF1*, *BNLF2a*, *BALF4* and *BLLF1* transcripts (A). Values shown are represented as expression relative to their maximum. Protein samples harvested from the same time points were subjected to western blot analysis, where samples were probed with antibodies to the indicated lytic cycle antigens (B).

Following induction of EBV replication in the AKBM cells, RNA samples were harvested over 48 hours post-induction (pi). qRT-PCR analysis was conducted on the two immediate early genes (*BZLF1* and *BRLF1*), two representative early genes (*BMLF1* and *BNLF2a*) and two representative late genes (*BLLF1* (encoding gp350) and *BALF4* (encoding gp110)). Upon induction, immediate early gene expression (*BZLF1* and *BRLF1*) occurred very rapidly with an increase in transcripts observed 1 hr pi, followed by peak expression at 2–3 hours pi (Figure 6A, upper panel). Transcripts for these two immediate early genes did not disappear completely after their peak expression, however *BZLF1* decreased quickly to low levels consistent with previous findings [12]. There were still more than 40% of the maximal *BRLF1* transcripts present 24 hours pi compared to only 5% of the maximal *BZLF1* transcripts at the same time point. Early gene message was expressed rapidly after induction with both *BMLF1* and *BNLF2a* reaching their peak expression at 4 hours pi (Figure 6A, middle panel). However, *BMLF1* message decreased quickly over the next 8 hours almost to its final levels, while high relative levels of *BNLF2a* message were maintained over the next 20 hours from peak expression dropping to 40% of the maximal level by 48 hours pi. As expected, induction of the late gene *BALF4* and *BLLF1* transcripts was slower, with peak expression at 12 hours and 24 hours, respectively (Figure 6A, lower panel).

We next turned to examine the protein expression kinetics in lytically induced AKBM cells by western blot analysis, employing antibodies specific to proteins used in our recognition assays where available (Figure 6B). Protein from each of the genes that had been measured by qRT-PCR was detected shortly following the expression of the corresponding transcript. Thus BZLF1, BRLF1 and BMLF1 protein were clearly detected at 2 hours pi as was another early protein BALF2. BNLF2a protein was also weakly detected at this point and clearly detected at 3 hours pi. BMRF1 showed delayed protein expression kinetics, being detected at 3–4 hours pi. Expression of the protein levels remained mostly stable for the duration of the time course, with the exception of BNLF2a which was lost from the cells at 12–48 hours pi. The late protein BALF4 was expressed by 6 hours and increased with time, while a second representative late protein, BFRF3, showed much delayed expression kinetics.

### Surface HLA class I levels remain unaltered in the immediate early/early phases of lytic cycle in ΔBNLF2a LCLs, yet are downmodulated during late lytic cycle

**Figure 7:**
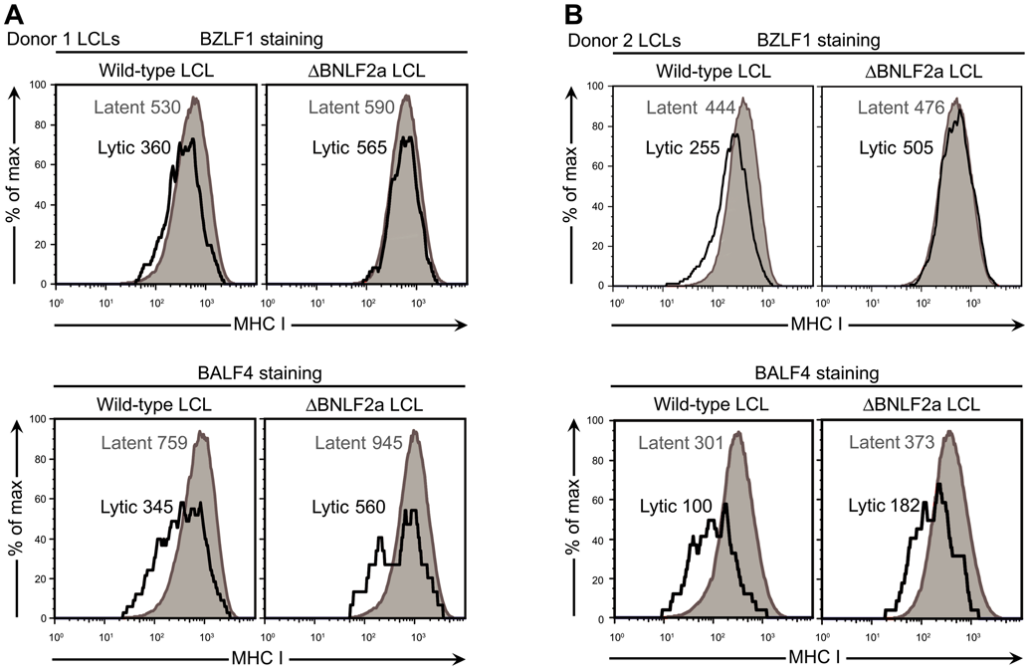
Surface HLA-class I expression in wild-type and ΔBNLF2a LCLs expressing immediate early or late antigens. Wild-type and ΔBNLF2a LCLs were stained for surface HLA-class I and expression levels measured by flow cytometry on cells co-stained for lytic antigens: either the immediate early antigen BZLF1 (upper panels), or the late antigen BALF4 (lower panels). The panels show histograms and MFI values of cell surface HLA-class I expression gated on cells with latent virus (lytic antigen negative, shaded histogram) or lytic virus (lytic antigen positive, open histogram). Staining data is presented from (A) Donor 1 LCLs and (B) Donor 2 LCLs.

The results from our recognition experiments indicated that the deletion of BNLF2a did not lead to any increase in recognition of late antigens by their cognate CD8^+^ T cells. Interestingly these late proteins were expressed when protein levels of BNLF2a were declining to low levels. Potentially other immune evasion proteins may be active at these later time points, preventing efficient presentation of epitopes to CD8^+^ T cells. To explore this possibility we performed flow cytometric analysis of surface HLA class I levels on wild-type and ΔBNLF2a LCLs from different donors, which had been co-stained for viral proteins expressed at different phases of lytic cycle. Wild-type LCLs stained for BZLF1 expression showed a decrease in surface HLA class I levels by around 1/3 of the level in latent (lytic antigen negative) cells, yet BZLF1 expressing ΔBNLF2a LCLs showed little to no decrease in surface HLA class I levels (Figure 7A and B upper panels). However, when cells were stained for the late lytic cycle protein BALF4, surface HLA class I levels in both the wild-type and ΔBNLF2a LCLs were decreased by around half of the level of that seen in latent cells (Figure 7A and B lower panels).

## Discussion

In this study we have shown that CD8^+^ T cell recognition of immediate early and early lytic cycle antigens is dramatically increased in LCLs transformed with a mutant EBV lacking the immune evasion gene *BNLF2a* compared to the recognition of wild-type EBV transformed LCLs. This increase in recognition was conserved across different HLA-class I backgrounds and these effects were seen using multiple different CD8^+^ T cell specificities, reinforcing the role of BNLF2a in active immune evasion during EBV lytic cycle replication. No observable difference in recognition of late lytic cycle antigens was observed, and peptide titration analysis of the late-specific CD8^+^ T cell clones ruled out the possibility that these effectors were simply less avid than those specific for the immediate early and early phases.

The observed increase in recognition of immediate early antigens was not anticipated when considered in the light of *BNLF2a's* previously described expression kinetics, where *BNLF2a* transcripts were not found to peak until at least 4 hours after immediate early gene expression [13]. By performing detailed analysis of the transcription and protein expression kinetics of *BNLF2a* and the immediate early genes in an EBV-infected B cell line in which lytic replication could be induced, we found that although immediate early protein expression was initiated prior to that of BNLF2a, there was a substantial increase in the expression immediate early proteins coincident with the expression of BNLF2a at 3 hours post induction. Epitopes derived from the first wave of immediate early protein synthesis will have no protection from being processed and presented to CD8^+^ T cells. However given that the major source of epitopes feeding the class I antigen processing pathway is now thought to be from *de-novo* synthesized proteins in the form of short-lived defective ribosomal products (DRiPs) rather than long lived protein (reviewed in [14]), expression of BNLF2a during this second wave of expression of the immediate early proteins would restrict the supply of epitope peptides at this time.

Analysis of the sequence of early protein expression using the inducible lytic replication system showed that BNLF2a was expressed with the first wave of early proteins, BALF2 and BMLF1. Similar to what is seen with the immediate early proteins, BNLF2a's expression was upregulated coincident with the increasing expression of these early proteins, again at a time when epitope production from these proteins is likely to be maximal. T cell recognition experiments using effectors specific for these proteins showed that deletion of BNLF2a from the targets caused clear increases in recognition of epitopes derived from these proteins compared to those expressed in wild-type targets. This indicates that although BNLF2a is expressed coincidently with these proteins, it can afford a substantial degree of protection from T cell recognition at this stage. Consistent with this finding was the observation that BNLF2a-deficient cells expressing BZLF1, and thus including those cells progressing through to early stages of the replicative cycle, showed an increase in class I MHC levels relative to wild type transformed cells, confirming BNLF2a's role in inhibiting antigen presentation at this time.

When different CD8^+^ T cell specificities were assayed for their ability to recognize their cognate antigen presented by the ΔBNLF2a LCLs as compared to the wild-type LCLs, variable levels of increased recognition were seen for the different T cell specificities. In some cases why this variability occurs is not clear. The abundance of the source protein does not appear to play a role as T cells specific for the three epitopes derived from BRLF1 namely AEN, YVL and IACP show quite different levels of increased recognition of the ΔBNLF2a LCL. The TAP dependence of the epitopes studied where determined does not appear to correlate with recognition. Furthermore as the hydrophobicity of peptides broadly correlates with the TAP independence [11], no clear correlation is seen between the hydrophobicity or likely TAP independence and the increase in recognition. The HLA C presented epitope IACP was consistently more greatly recognized when presented by the ΔBNLF2a LCL compared to other epitopes presented from these LCLs. Some immune evasion proteins have been described to have allele specificity, such as the cytomegalovirus encoded US3 protein [15], however whether BNLF2a shows allele-specificity requires further investigation.

When the expression profile of the early protein BMRF1 was examined it showed a delayed pattern of expression relative to BNLF2a and the other early proteins studied. T cell recognition assays with clones specific to epitopes derived from BMRF1 consistently showed the lowest increase in recognition by T cells in BNLF2a-deficient targets, indicating that BNLF2a has some but perhaps a lesser effect on presentation of epitopes from this protein. This raises the possibility that other mechanisms are preventing effective antigen presentation during this later phase of early gene expression. More compelling evidence for other EBV-encoded class I evasion mechanisms comes from the study of the T cell recognition and expression kinetics of late phase protein targets. The expression of the best characterized late protein, BALF4, was seen to increase in the inducible cell line from 6 hours post induction, with heightened expression occurring at 8–12 hours. At this stage BNLF2a protein levels were decreasing in these cells, yet T cell recognition experiments using late-specific effectors to BALF4 and BILF2 show very poor recognition of wild-type LCL targets. Importantly however, when using the same late-specific effectors in recognition assays of BNLF2a-deleted targets, no increase in detection is seen compared to wild-type targets. Given that the target of BNLF2a is the TAP complex and we have shown previously that this complex is not degraded during EBV lytic cycle replication, at least at 24 hours post-induction of lytic cycle [8], this would suggest that EBV-encoded mechanisms other than BNLF2a are operating to block antigen presentation during the late phase of replication. Supporting this idea is the observation that BNLF2a-deficient LCLs expressing the late antigen BALF4 show decreased levels of surface class I MHC molecules similar to wild-type virus transformed cells.

Evasion of CD8^+^ T cell recognition is likely to be most efficient when multiple points of the antigen processing pathway are targeted, with BNLF2a being one of potentially several immune evasion proteins. Other proteins potentially involved in this process include the early-expressed gene *BGLF5* which functions as an alkaline exonuclease and a host protein synthesis inhibitor. BGLF5's inhibition of global protein synthesis, including that of class I MHC, can inhibit effective CD8^+^ T cell recognition of cognate targets [16],[17]. A second candidate recently identified in modulating surface class I levels is the early phase expressed gene *BILF1*, whose product acts to promote turnover of surface class I molecules [18]. Conceivably these proteins may act in a complementary manner to BNLF2a at early time points, initially by BILF1 clearing class I complexes containing immediate early epitopes from the surface of the cell that were produced before BNLF2a function was established and then BGLF5 acting to prevent effective class I synthesis.

As to BNLF2a's function *in vivo*, it is difficult to draw direct inferences from animal herpesvirus models in which immune evasion genes have been disrupted since the viruses used, either the β-herpesvirus murine cytomegalovirus (MCMV) or the γ-2 herpesvirus MHV-68, have different *in vivo* infection biology compared to EBV. Nevertheless, recent work on the β-herpesvirus MCMV has indicated that deletion of viral regulators of antigen processing either has no effect on immunodominance hierarchies or virus loads [19],[20], or surprisingly, decreases the size of at least some CD8^+^ T cell reactivities [21]; perhaps as a consequence of increased antigen clearance. In the case of MHV-68 which has a similar cellular tropism to EBV, deletion of the immune evasion gene *mK3*, which is expressed during latency establishment and also during lytic replication, led to increased CD8^+^ T cell responses to lytic proteins yet had little effect on levels of virus undergoing lytic replication. It did however decrease latent viral loads, suggesting a role for mK3 in amplifying the latent virus reservoir [22]. By contrast, BNLF2a is not expressed during latency and EBV's mechanism of amplifying the latent viral load may come more from its growth transforming ability, by directly expanding latently infected B cells when first colonizing the B cell system. Ultimately, the impact BNLF2a has on immunodominance, viral loads and transmission may be best addressed using the closely related rhesus macaque lymphocryptovirus (Cercopithicine herpesvirus 15) model. This virus has a similar biology to EBV and the same repertoire of genes [23], including a BNLF2a homologue which has the ability to cause surface class I MHC downregulation when expressed in rhesus cell lines [9].

Overall, these results indicate that BNLF2a functions to protect the immediate early and early proteins from being efficiently processed and presented to CD8^+^ T cells. We would expect then that *in vivo* BNLF2a would function to shield virus reactivating from latency or initiating lytic cycle replication. Such stage-specific expression of immune evasion genes is a feature of several herpesviruses. Perhaps the clearest example comes from CMV where multiple proteins involved in disrupting CD8^+^ T cell recognition of infected cells have been described. During CMV replication the US3 gene, whose product retains class I complexes in the endoplasmic reticulum, is abundantly expressed during the immediate early phase [24]–[26], while the gene US11, whose product dislocates class I molecules from the endoplasmic reticulum into the cytosol, is expressed predominantly during early phase replication, and the TAP inhibitor US6 is transcribed in early and late phases [27]. The differential expression of these genes then may be in part why these viruses utilize multiple evasion mechanisms. In the case of EBV replication, as BNLF2a acts in a stage-specific manner we suggest that it will act in concert with other EBV encoded immune evasion genes to reduce efficient T-cell surveillance of reactivating or productively infected host cells.

## Materials and Methods

### Ethics statement

All experiments were approved by the South Birmingham Local Research Ethics Committee (07/Q2702/24). All patients provided written informed consent for the collection of blood samples and subsequent analysis.

### Recombinant EBV strains

Wild-type and ΔBZLF1 recombinant EBV BACs used have been previously described [10].The generation of a recombinant EBV BAC deleted for *BNLF2a* was performed as follows: a targeting vector containing the *BNLF2a* region was used to delete *BNLF2a* from the wild-type B95.8 EBV BAC genome. The introduction of a tetracycline cassette, flanked by FLP recombinase target sites (*FRT*), between a unique *Xho*I site (−6 bp from the *BNLF2a* open reading frame ATG initiation codon) and *Aat*II site (108 bp downstream of the *BNLF2a* initiation codon) allowed for the insertional mutagenesis of the *BNLF2a* ORF. This left a 66 bp 3′ *BNLF2a* sequence fragment intact that was lacking an initiation codon. Homologous recombination of the target vector, via flanking sequences either side of the truncated *BNLF2a*, allowed for the introduction of the mutation into the wild-type EBV B95.8 BAC sequence. Successfully recombined clones were doubly selected on tetracycline and chloramphenicol (the latter resistance cassette present in the wild-type backbone sequence), followed by removal of the tetracycline cassette through transformation of an FLP recombinase. Bacterial clones that survived this selection process were screened with several restriction enzymes and also sequenced to confirm successful disruption of *BNLF2a* (data not shown).

Wild-type, ΔBNLF2a and ΔBZLF1 recombinant virus preparations were generated by stably transfecting 293 cells with the corresponding EBV BAC genome and inducing lytic cycle replication, as previously described [10],[28].

### Generation of target cell lines and T cell clones

B lymphoblastoid target cell lines (LCLs) were generated by transformation of laboratory donor B lymphocytes (isolated by positive CD19 Dynabead® (Invitrogen) selection, as per the manufacturer's instructions) with the following recombinant EBV viruses: wild-type, ΔBNLF2a and ΔBZLF1. LCLs were maintained in standard medium (RPMI-1640, 2 mM glutamine, and 10% [vol/vol] FCS). Effector CD8^+^ T cells were generated as previously described [6],[29]. CD8^+^ T cell clones used in this study were specific for the following epitopes derived from the respective EBV gene products: RAKFKQLL from BZLF1 presented by HLA-B*0801 [30], AENAGNDAC from BRLF1 presented by HLA-B*4501 [6], IACPIVMRYVLDHLI from BRLF1 presented by HLA-C*0202 [6], ARYAAYYLQF from BALF2 presented by HLA-B*2705 [6], TLDYKPLSV from BMRF1 presented by HLA-A*0201 [31], FLDKGTYTL from BALF4 presented by HLA-A*0201 [6], RRRKGWIPL from BILF2 presented by HLA-B*2705 [6], YVLDHLIVV from BRLF1 presented by HLA-A*0201 [32], GLCTLVAML from BMLF1 presented by HLA-A*0201 [29],[33].

### CD8^+^ T cell recognition experiments

The capacity of lytic-specific CD8^+^ T cell clones to recognize lytically replicating cells within LCLs of the relevant HLA type was measured by IFNγ ELISA (Endogen). Briefly, target LCLs (5×10^4^ cells/well) were co-cultured in triplicate with effector CD8^+^ T cells (5×10^3^ cells/well) in V-bottomed 96-well plates in a total of 200 µl standard media/well and incubated overnight at 37°C with 5% CO_2_. After 18 hours 50 µl of culture supernatant from each well was used for IFNγ detection by ELISA

### Reactivation of AKBM cells into EBV lytic cycle

AKBM cells and their use have been described previously [8]. Briefly, this EBV infected cell line contains a reporter GFP-rat CD2 construct under the control of an early EBV promoter to allow identification of cells in lytic cycle. Prior to induction, AKBM cells were sorted by FACS to exclude any GFP+ve cells that had spontaneously entered lytic cycle. The GFP-ve fraction was then induced into lytic cycle by crosslinking of surface IgG molecules as previously described [8]. Cells were then harvested at the indicated timepoints post induction for western blotting and qRT-PCR analysis.

### Western blot assays

Total cell lysates were generated by denaturation in lysis buffer (final concentration: 8 M urea, 50 mM Tris/HCl pH 7.5, 150 mM sodium 2-mercaptoethanesulfonate) and sonicated. Protein concentration was determined using a Bradford protein assay (Bio-Rad), and 20 µg of protein for each sample was separated by SDS-polyacrylamide gel electrophoresis (SDS-PAGE) using a Bio-Rad Mini Gel tank. Proteins were blotted onto nitrocellulose membranes and blocked by incubation for 1 hr in 5% skimmed-milk powder dissolved in PBS-Tween 20 detergent (0.05% [vol/vol]). Specific proteins were detected by incubation with primary antibodies for BZLF1 (murine monoclonal antibody (MAb) BZ.1, final concentration 0.5 µg/ml, [34]), BRLF1 (murine MAb clone 8C12, final concentration 2.5 µg/ml, Argene, cat. # 11-008), BMLF1 (rabbit serum to EBV BSLF2/BMLF1-encoded SM, clone EB-2, used at 1/6000 [35]), BMRF1 (murine MAb clone OT14-E, used at 1/2000 [36]), BALF2 (murine MAb clone OT13B, used at 1/5000, [37]), BNLF2a (clone 5B9, used at 1/100, a rat hybridoma supernatant directed to the N-terminal region of BNLF2a generated by E. Kremmer through immunization of Lou/C rats with KLH-coupled BNLF2a peptides, followed by fusion of rat immune spleen cells with the myeloma cell line P3X63-Ag8.653), BALF4 (murine Mab clone L2, used at 1/100, [38]) BFRF3 (rat MAb clone OT15-E, used at 1/250, J. M. Middeldorp, [39]) and EBNA2 (murine MAb clone PE-2, used at 1/50, [40]) for 2 hrs at room temperature, followed by extensive washes with PBS-Tween. Detection of bound primary antibodies was by incubation for 1 hr with appropriate horseradish peroxidase (HRP)-conjugated secondary antibodies (goat anti-mouse IgG:HRP (Sigma, cat. #A4416), goat anti-rat IgG:HRP (Sigma, cat. #A9037), and goat anti-rabbit IgG:HRP (Sigma, cat. #A6154). Bound HRP was then detected by enhanced chemiluminescence (ECL, Amersham).

### Quantitative real-time reverse transcription PCR

Total RNA was extracted from 0.5×10^6^ cells using a NucleoSpin® RNA II kit (Machery-Nagel) followed by Turbo DNA-free™ (Ambion/Applied Biosystems) treatment to remove any residual DNA contamination, as per the manufacturers' instructions. 500 ng of RNA was reverse transcribed into cDNA using a pool of primers specific for *BZLF1*, *BRLF1*, *BMLF1*, *BNLF2a*, *BALF4* and *BLLF1*, with *GAPDH* included as an internal control, followed by subsequent quantitative-PCR (q-PCR). EBV lytic gene primers were as follows (primer sequences in parenthesis): BZLF1 (**cDNA** 5′GCAGCCACCTCACG3′, **F** 5′ACGACGCACACGGAAACC3′, **R** 5′CTTGGCCCGGCATTTTCT3′, **probe** 5′GCATTCCTCCAGCGATTCTGGCTGTT3′), BRLF1 (**cDNA** 5′CAGGAATCATCACCCG3′, **F** 5′TTGGGCCATTCTCCGAAAC3′, **R** 5′TATAGGGCACGCGATGGAA3′, **probe** 5′AGACGGGCTGAGAATGCCGGC3′), BMLF1 (**cDNA** 5′GAGGATGAAATCTCTCCAT3′, **F** 5′CCCGAACTAGCAGCATTTCCT3′, **R** 5′GACCGCTTCGAGTTCCAGAA3′, **probe** 5′AACGAGGATCCCGCAGAGAGCCA3′), BNLF2a (**cDNA** 5′GTCTGCTGACGTCTGG3′, **F** 5′TGGAGCGTGCTTTGCTAGAG3′, **R** 5′GGCCTGGTCTCCGTAGAAGAG3′, **probe** 5′CCTCTGCCTGCGGCCTGCC3′), BALF4 (**cDNA** 5′CCATCAACAGGCCCTC3′, **F** 5′CCAGCTTTCCTTTCCGAGTCT 3′, **R** 5′ACACTGGATGTCCGAGGAGAA3′, **probe** 5′TCCAGCCACGGCGACCTGTTC3′), and BLLF1 (**cDNA** 5′ACTGCAGTACTAGCATGG3′, **F** 5′AGAATCTGGGCTGGGACGTT3′, **R** 5′ACATGGAGCCCGGACAAGT3′, **probe** 5′AGCCCACCACAGATTACGGCGGT3′). cDNA and forward/reverse primers were synthesised by Alta Bioscience (University of Birmingham). Probes were synthesised by Eurogentec S.A and labelled with 5′ FAM fluorophore and 3′ TAMRA quencher. Data was normalised to *GAPDH* expression, and expressed as relative to the maximal level of transcript for each gene.

### Flow cytometry

LCLs were assayed for the percentage of cells spontaneously reactivating into lytic cycle by intracellular staining for BZLF1. Cells were first fixed using 100 µl of Ebiosciences Intracellular (IC) Fixative (cat. # 00-8222-49) for 1 hr on ice, followed by permeabilisation through the addition of 100 µl Triton X-100 (final concentration 0.2%) and a further 30 minute incubation on ice. After extensive washing with PBS, cells were incubated with 1 µg/ml of either MAb BZ.1 (anti-BZLF1) or with an IgG_1_ isotype control antibody for 1 hr at 37°C. Cells were washed twice in PBS and then incubated with 1∶20-diluted R-phycoerythrin-conjugated goat anti-mouse IgG_1_ antibody (AbD Serotec, cat. # STAR132PE) for 1 hr at 37°C. Following further washes cells were resuspended in IC fixative and analysed on a Dako Cyan flow cytometer (Dako, Denmark).

LCL surface HLA class I and intracellular lytic-cycle EBV antigens were detected simultaneously by first staining viable cells with 1∶15-diluted allophycocyanin-conjugated-anti-human HLA-A,B,C (Biolegend, cat. # 311410) antibody for 30 minutes on ice. Cells were then washed extensively in PBS and fixed and permeabilised as above, followed by incubation for 1 hr at 37°C with 1 ug/ml of either MAb BZ.1 (immediate early antigen BZLF1) or L2 (late antigen BALF4), or IgG_1_ isotype control. After several washes in PBS cells were incubated for 1 hr with 1∶20-diluted R-phycoerythrin-conjugated goat anti-mouse IgG_1_ antibody as above. Cells were washed and fixed as above, followed by analysis on a Dako cytometer (Dako, Denmark). All flow data was analyzed using FlowJo software (Tree Star).

## Supporting Information

Figure S1 T cell recogntion of ΔBNLF2a LCLs when BNLF2a is expressed in these cells. ΔBNLF2a LCLs were transfected by electroporation with a plasmid which co-expressed BNLF2a and the truncated nerve growth factor (NGFR) gene. After 48 hours, BNLF2a expressing cells were purified by selecting NGFR expressing cells. These cells were used in standard T cell recognition assays in parallel with the NGFR-negative cells from the transfection, wild-type virus transformed LCLs, the unmanipulated ΔBNLF2a LCL and the ΔBZLF1 knock out LCL. CD8^+^ T cells specific for the immediate early epitope AEN and early epitope ARYA were used as effectors in parallel assays. One representative assay of two transfection experiments is shown.
(0.71 MB PDF)
