# Supplementary figures and images for: Stage-Specific Inhibition of MHC Class I Presentation by the Epstein-Barr Virus BNLF2a Protein during Virus Lytic Cycle
Source: PLoS Pathog. 2009 Jun 26;5(6):e1000490. doi: 10.1371/journal.ppat.1000490 (PMC2695766; doi:10.1371/journal.ppat.1000490)

Donor 4 LCLs < <sup>wt</sup> 0.5% lytic  
<sup>Δ2a</sup> 0.5% lytic

### BRLF1 AEN / B\*4501

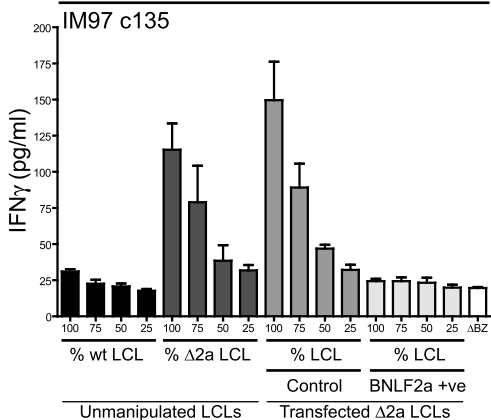

### BALF2 ARYA / B\*2705

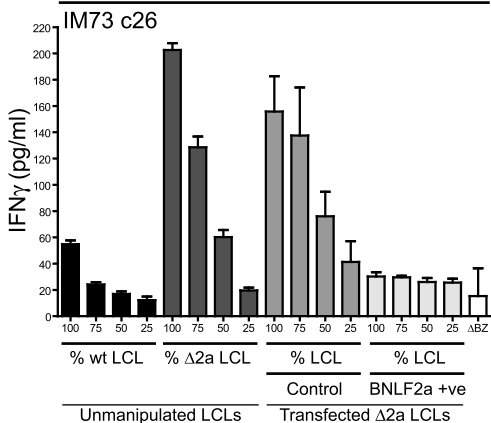

Supplement: Figure S1 — T cell recogntion of ΔBNLF2a LCLs when BNLF2a is expressed in these cells. ΔBNLF2a LCLs were transfected by electroporation with a plasmid which co-expressed BNLF2a and the truncated nerve growth factor (NGFR) gene. After 48 hours, BNLF2a expressing cells were purified by selecting NGFR expressing cells. These cells were used in standard T cell recognition assays in parallel with the NGFR-negative cells from the transfection, wild-type virus transformed LCLs, the unmanipulated ΔBNLF2a LCL and the ΔBZLF1 knock out LCL. CD8+ T cells specific for the immediate early epitope AEN and early epitope ARYA were used as effectors in parallel assays. One representative assay of two transfection experiments is shown. (0.71 MB PDF) [file ppat.1000490.s001.pdf]
